# A pathway-directed positive growth restoration assay to facilitate the discovery of lipid A and fatty acid biosynthesis inhibitors in *Acinetobacter baumannii*

**DOI:** 10.1371/journal.pone.0193851

**Published:** 2018-03-05

**Authors:** Daryl L. Richie, Lisha Wang, Helen Chan, Gianfranco De Pascale, David A. Six, Jun-Rong Wei, Charles R. Dean

**Affiliations:** Novartis Institutes for BioMedical Research, Emeryville, CA, United States of America; University of Cambridge, UNITED KINGDOM

## Abstract

*Acinetobacter baumannii* ATCC 19606 can grow without lipooligosaccharide (LOS). Lack of LOS can result from disruption of the early lipid A biosynthetic pathway genes *lpxA*, *lpxC* or *lpxD*. Although LOS itself is not essential for growth of *A*. *baumannii* ATCC 19606, it was previously shown that depletion of the lipid A biosynthetic enzyme LpxK in cells inhibited growth due to the toxic accumulation of lipid A pathway intermediates. Growth of LpxK-depleted cells was restored by chemical inhibition of LOS biosynthesis using CHIR-090 (LpxC) and fatty acid biosynthesis using cerulenin (FabB/F) and pyridopyrimidine (acetyl-CoA-carboxylase). Here, we expand on this by showing that inhibition of enoyl-acyl carrier protein reductase (FabI), responsible for converting trans-2-enoyl-ACP into acyl-ACP during the fatty acid elongation cycle also restored growth during LpxK depletion. Inhibition of fatty acid biosynthesis during LpxK depletion rescued growth at 37°C, but not at 30°C, whereas rescue by LpxC inhibition was temperature independent. We exploited these observations to demonstrate proof of concept for a targeted medium-throughput growth restoration screening assay to identify small molecule inhibitors of LOS and fatty acid biosynthesis. The differential temperature dependence of fatty acid and LpxC inhibition provides a simple means by which to separate growth stimulating compounds by pathway. Targeted cell-based screening platforms such as this are important for faster identification of compounds inhibiting pathways of interest in antibacterial discovery for clinically relevant Gram-negative pathogens.

## Introduction

The relentless emergence of antibacterial resistance has led to a resurgence in public and private research to discover novel antibiotics, in particular for Gram-negative infections due to the paucity of compounds in clinical development [1]. The Gram-negative cell envelope consists of a double membrane organized as a phospholipid (PL) inner membrane (IM) bilayer and an asymmetric outer membrane (OM) comprised of a PL inner leaflet and an outer leaflet composed primarily of lipopolysaccharide (LPS) [2]. The Gram-negative double membrane structure provides an effective barrier to the penetration of hydrophobic molecules due to LPS and to hydrophilic molecules due to the phospholipid bilayer [3]. This presents challenges in antibacterial drug discovery for Gram-negative pathogens, because many potent enzyme inhibitors cannot accumulate sufficiently in cells to exert growth inhibitory effects [4]. This has led to an interest in inhibiting enzymes important for envelope biosynthesis, such as those mediating LPS or fatty acid biosynthesis. Inhibitors of such targets could be stand-alone antibacterials in the case of targets that are essential for growth (e.g. LpxC inhibitors), or could be suited to combination approaches since inhibition of cell envelope targets may also affect the permeability barrier and potentiate the cellular activity of other antibacterials [2, 5].

**Fig 1:**
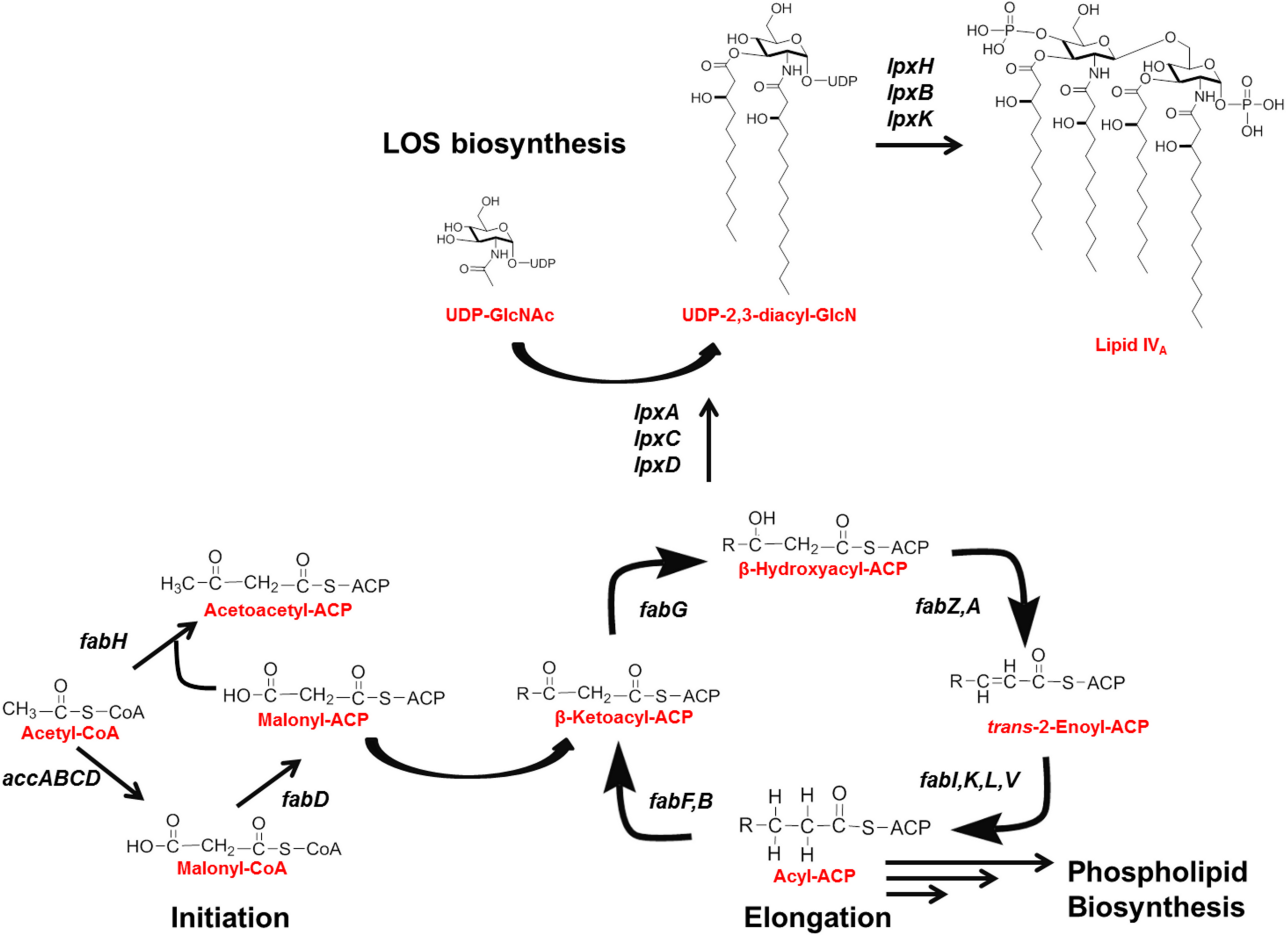
Schematic of predicted lipid A and FASII biosynthetic pathway in *A*. *baumannii* ATCC 19606.

Many of the enzymes involved in LPS biosynthesis and transport are essential and conserved across a diverse range of Gram-negative pathogens. Correspondingly, targeting LPS assembly remains an area of interest for the development of novel antibacterials [6–10]. In *E*. *coli*, the best-studied organism, LPS biogenesis is initiated by three soluble enzymes LpxA, LpxC and LpxD, which add two β-hydroxyacyl chains to UDP-*N*-acetylglucosamine (UDP-GlcNAc) forming UDP-2,3-diacyl-GlcN [10–16]. Of the LPS initiation steps, the area of most intense emphasis for drug development is LpxC, a Zn^2+^-dependent deacetylase and the first committed step in lipid A biosynthesis. LpxC catalyzes the deacetylation of UDP-3-*O*-(R-3-hydroxyacyl)GlcNAc to produce UDP-3-*O*-(R-3-hydroxylacyl)GlcN. LpxD then catalyzes the addition of a second β-hydroxyacyl chain from acyl-ACP generating UDP-2,3-diacyl-GlcN[17–24]. Next, UDP-2,3-diacyl-GlcN is hydrolyzed by LpxH, forming lipid X, and LpxB then catalyzes the condensation of UDP-2,3-diacyl-GlcN and lipid X to form the tetraacylated disaccharide 1-monophosphate (DSMP) [25, 26]. Lipid IV_A_ is then formed through phosphorylation of DSMP by the integral membrane kinase LpxK at the 4' position [27] (Fig 1).

To complete the formation of mature LPS, inner core sugars are added to lipid IV_A_ along with secondary acyl chains via LpxL and LpxM to generate core-lipid A [28]. Core-lipid A is subsequently flipped across the IM by MsbA [29, 30], where it is decorated with O-antigen polysaccharide polymers, and then mature LPS is transported across the OM and presented at the cell surface by the ATP dependent lipopolysaccharide transport (Lpt) system [31–33]. Within the Lpt system, the LptB_2_FG complex extracts the LPS from the inner membrane and a bridge is formed via LptC, LptA, and the N-terminus of LptD which facilitates LPS movement across the periplasmic space [34]. LptD forms a complex with LptE [35, 36], which is responsible for translocating LPS across the outer membrane and insertion into the outer leaflet via a lateral gate opening mechanism [37–40]. Some Gram-negatives, such as *A*. *baumannii*, appear to lack a dedicated O-antigen ligase and do not attach an O-antigen to the lipid A core, thus producing lipooligosaccharide (LOS) [41–44]. The minimal structure needed for viability of *E*. *coli* in laboratory conditions is lipid IV_A_ [45], however, this differs among Gram-negatives, because in *P*. *aeruginosa* phosphorylation (WaaP) of core oligosaccharides is required for growth [46–48] and some species including *A*. *baumannii* are able to survive, at least in laboratory conditions, in the absence of LOS biosynthesis or transport [49–57].

The IM of the Gram-negative cell envelope is comprised of PL generated through the bacterial type II fatty acid synthesis (FASII) pathway. The first and committed step is the biotin-dependent carboxylation of acetyl-CoA to produce malonyl-CoA by the multi-subunit complex acetyl-CoA carboxylase (ACC) [58]. Initiation of fatty acid biosynthesis continues with the conversion of malonyl-CoA to malonyl-ACP by FabD followed by the condensation of malonyl-ACP with acetyl-CoA to generate acetoacetyl-ACP by FabH [58]. From here, a repeating elongation process begins that adds 2 carbons per cycle through the elongation enzymes of FabG, FabZ and FabI, followed by additional elongation rounds initiated by FabF through the condensation of malonyl-ACP with acyl-ACP [59] (Fig 1).

Fatty acid biosynthesis is also an area of interest for drug development with efforts focused around rate-determining reactions, including FabB/F, FabH, enoyl-ACP reductases (FabI) and ACC [59, 60]. However, the ability of some Gram-positive pathogens to bypass inhibition of de novo fatty acid biosynthesis through uptake of exogenous fatty acids from the media [60–62] and the presence of different enzyme isoforms performing the same reaction in several key steps along the FASII pathway (enoyl-ACP reductase) [63–65] suggests that targeting of fatty acid biosynthesis would be best suited for narrow spectrum drug development [59]. Nevertheless, the clinical development of enoyl-ACP reductase inhibitors for the treatment of acute bacterial skin and skin structure infection (ABSSSI) caused by methicillin-resistant *Staphylococcus aureus* (MRSA) including CG400549 (CrystalGenomics) [66–68] and afabicin (Debio1450, Debiopharm) [69, 70] continues to be investigated.

The importance for bacteria to balance LPS and PL biosynthesis to maintain envelope function and integrity has long been recognized in *E*. *coli* where regulatory mechanisms include negative feedback loops, shared substrates, and protease-controlled degradation of LPS biosynthesis enzymes in order to maintain equilibrium between PL and LPS because imbalances can lead to cell death [71–76]. Given the tightly controlled balancing of LPS and PL biosynthesis necessary for *E*. *coli*, it is currently not fully understood how organisms such as *A*. *baumannii* and *Neisseria meningitidis* that can survive without LOS adapt to loss of LOS biosynthesis [52, 55, 59, 77]. Nevertheless, FASII has been shown to be essential in *Neisseria*, and bioinformatics predictions for *A*. *baumannii* suggest that the genomic organization of fatty acid, phospholipid, and LOS synthesis are similar to *E*. *coli*, such that FASII is also expected to be essential in *A*. *baumannii* [59, 77]. Furthermore, inhibiting targets such as LpxA or LpxC, which are not essential in some *A*. *baumannii* clinical isolates, may still provide an effective therapeutic approach, because the LOS-containing outer membrane is required for virulence and intrinsic drug resistance [78].

We previously demonstrated that down-regulation of *lpxK* in *A*. *baumannii* ATCC 19606 led to a toxic accumulation of LOS intermediates [79]. Furthermore, under LpxK depletion conditions the inhibition of LOS (LpxC) and fatty acid biosynthesis (FabB/F, ACC) could ameliorate growth defects through reducing the accumulation of toxic LOS intermediates [79]. In this study, we expand on our previous findings by showing that inhibition of FabI can also rescue growth of *A*. *baumannii* during LpxK depletion, and fatty acid inhibitor mediated rescue is not observed at 30°C. Finally, we exploited this phenomenon to develop a straightforward cell-based positive-growth screening platform useful for identification of lipid A or fatty acid biosynthesis inhibitors in the clinically-relevant pathogen *A*. *baumannii*.

## Materials and methods

### Bacterial strains and growth conditions

The bacterial strains used in this study were *A*. *baumannii* ATCC 19606 from the American Type Culture Collection (ATCC) and the constructed mutants *lpxC*::*Km*^R^, *lptD*::*Km*^R^, and JWK0013(pNOV044), an isopropyl β-d-1-thiogalactopyranoside (IPTG) regulated *lpxK* strain as previously described [56, 79]. Cells were routinely grown in Mueller-Hinton II (MHIIB) Broth (Cation-Adjusted) (3.0 g/L beef extract, 17.5 g/L acid hydrolysate of casein, 1.5 g/L starch, 20–25 mg/L calcium, 10–12.5 mg/L magnesium) or agar (Difco 225250).

To determine which antibiotics could rescue growth during LpxK depletion, strain JWK0013(pNOV044) was grown overnight at 37°C on Mueller-Hinton Agar (MHA), supplemented with 1 mM IPTG (Calbiochem). The following day, cells were suspended in 1 mL of MHIIB, collected by centrifugation at 10,000 ×*g*, and suspended in fresh MHIIB for a total of 3 washes to remove trace amounts of IPTG. After the final wash, cells were suspended in 5 mL of MHIIB and the OD_600_ was adjusted to 0.01. Next, 100 μL of the cell suspension were spread on a fresh MHIIB plate and allowed to dry. Sterile paper disks (BBL, 231039) were added to the center of the plates and inoculated with 10 μL of DMSO (Sigma), IPTG (1 mM) or the antibiotic of interest at 12.8 mg/mL. The plates were incubated at 30°C or 37°C for 24 to 72 h before images were taken using a BIO-RAD ChemiDoc^Tm^ XRS+ with Image Lab^Tm^ 3.0 software.

### Determination of antibiotic susceptibility

Test compounds were dissolved in DMSO at 12.8 mg/mL (100-fold higher than the final assay concentration of 128 μg/mL), and in a standard 96-well plate sequential 2-fold serial dilutions were made in DMSO from wells 11–2 (corresponding final assay concentration of 0.25–128 μg/mL), leaving well 1 as the DMSO vehicle control and well 12 empty to serve as a sterility control. Using a 12-channel electronic pipette, 1 μL of each 100× drug concentration, including the DMSO only control, were transferred into a new 96-well U-bottom plate (Greiner bio-one, 650162). To generate suspensions for susceptibility testing, cells from a frozen glycerol stock were streaked on MHA plates and incubated overnight at 37°C. The following day, cell suspensions were prepared in accordance with the BBL Prompt Inoculation System (with the modification that cells were initially suspended in MHIIB medium instead of the supplied saline solution to limit lysis of the Δ*lptD* mutant) and further diluted 1:100 in MHIIB. Next, 100 μL of this inoculum were added to the drug dilution plate for a final concentration of 0.5–128 μg/mL. Plates were then incubated for 18–24 hours at 37°C before MIC determination. Bacterial growth was evaluated by visual inspection of the 96-well plate aided by the use of a viewing mirror.

### Medium-throughput positive growth restoration assay

Strain JWK0013(pNOV044) was streaked on MHA, supplemented with 1 mM IPTG, and incubated overnight at 37°C. The following day the cells were suspended in fresh MHIIB and centrifuged at 10,000 ×*g*, the supernatant removed, and again suspended in fresh MHIIB medium. This was repeated 2 times for a total of 3 washes. The cells were then diluted to an OD_600_ of 0.01, and 1 μL of the inoculum was dispensed into each well of the 96-well assay ready plates (described below) using a Thermo Scientific Matrix WellMate and WellMate Disposble tubing assembly (small-bore needles, #201–30002). Plates were then incubated at 37°C for 24 to 48 h unstacked and the fluorescence read (545 nm excitation and 590 nm emission) on the SpectraMax M5 Microplate Reader using SoftMax® Pro version 5. Image creation was performed with Microsoft Excel 2010. To prepare the assay-ready plates, a master plate was generated by dissolving test compounds in DMSO at 12.8 mg/mL (100-fold higher than the final assay concentration of 128 μg/mL), and 2-fold serial dilutions were performed in a 96-well plate (Greiner 650162) in DMSO from wells 11–2 (corresponding final assay concentration of 0.25–128 μg/ml) leaving well 1 as the DMSO vehicle control and well 12 for a positive control (IPTG 1 mM). Next, 1 μL of each 100× drug concentration was stamped from the master plate into a new 96-well U-bottom plate (Greiner bio-one, 650162). MHA was then melted, cooled to 65°C, and supplemented with 10% alamarBlue^®^ (*v*/*v*) (BIO-RAD, BUF012B). The medium was then dispensed over the entire assay plate, allowed to cool, and placed at 4°C in the dark until use within 3 days.

## Results

### Inhibition of enoyl-ACP reductase (FabI) rescues growth of *A*. *baumannii* under LpxK depletion conditions

We previously demonstrated that LpxK depletion in *A*. *baumannii* ATCC 19606 led to toxic accumulation of lipid A pathway intermediates that prevented growth [79]. Consistent with this, fatty acid biosynthesis inhibitors, including cerulenin (FabB/F) and pyridopyrimidine (ACC) reduced this accumulation, restoring growth [79]. The condensation enzymes FabF, FabB, and FabH are required for acyl chain elongation during fatty acid biosynthesis and are considered desirable drug targets as they are rate-determining reactions [59]. FabH is responsible for the initiation of new acyl chains, ultimately determining how many fatty acids are made [80, 81]. Subsequent to initiation of fatty acid biosynthesis by FabH, each new cycle of 2 carbon acyl chain elongation is triggered by FabF, while FabB has a similar function but is essential for elongation of unsaturated fatty acids [59, 82–84].

**Fig 2:**
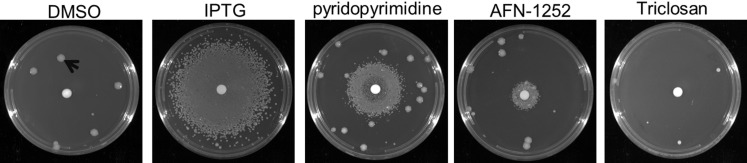
Inhibition of enoyl-ACP reductase (FabI) rescues growth of *A*. *baumannii* under LpxK depletion conditions. Growth of JWK0013(pNOV044) was not observed under noninducing conditions (DMSO, 10 μL per disk, arrow represents revertant and loss of IPTG regulation); growth of JWK0013(pNOV044) was restored in the presence of IPTG (10 μL @ 1 mM per disk); JWK0013(pNOV044) grew under noninducing conditions in the presence of pyridopyrimidine (10 μL @ 12.8 mg/mL per disk, ACC) and AFN-1252 (10 μL @ 3.2 mg/mL per disk, FabI) but not triclosan (10 μL @ 12.8 mg/mL per disk, FabI).

FabI is an enoyl-ACP reductase that catalyzes the last reductive step in the fatty acid biosynthetic cycle converting *trans*-2-enoyl-ACP into acyl-ACP. This step is also rate limiting, however, FabI is considered a pathogen-specific drug target due to the existence of redundant isoforms including FabL, FabK, and FabV [59]. Previously, we have shown growth rescue of *A*. *baumannii* ATCC 19606 under LpxK depletion conditions through inhibition of rate-limiting initiation steps at ACC, and FabB/F [79]. Here, we determined whether inhibition of FabI, which is not an initiation step but rather is responsible for pulling cycle elongation to completion, also caused growth rescue by testing AFN-1252 (Debio 1452), an inhibitor of *S*. *aureus* FabI [85, 86] (Fig 2). A sub-lethal concentration of AFN-1252 restored growth under LpxK depletion conditions, however, growth restoration was not observed in the presence of the FabI inhibitor triclosan at the concentrations tested, a phenomenon that is currently not fully understood.

### Inhibitors of fatty acid biosynthesis can be distinguished from LpxC inhibitors through temperature differentiated rescue

**Fig 3:**
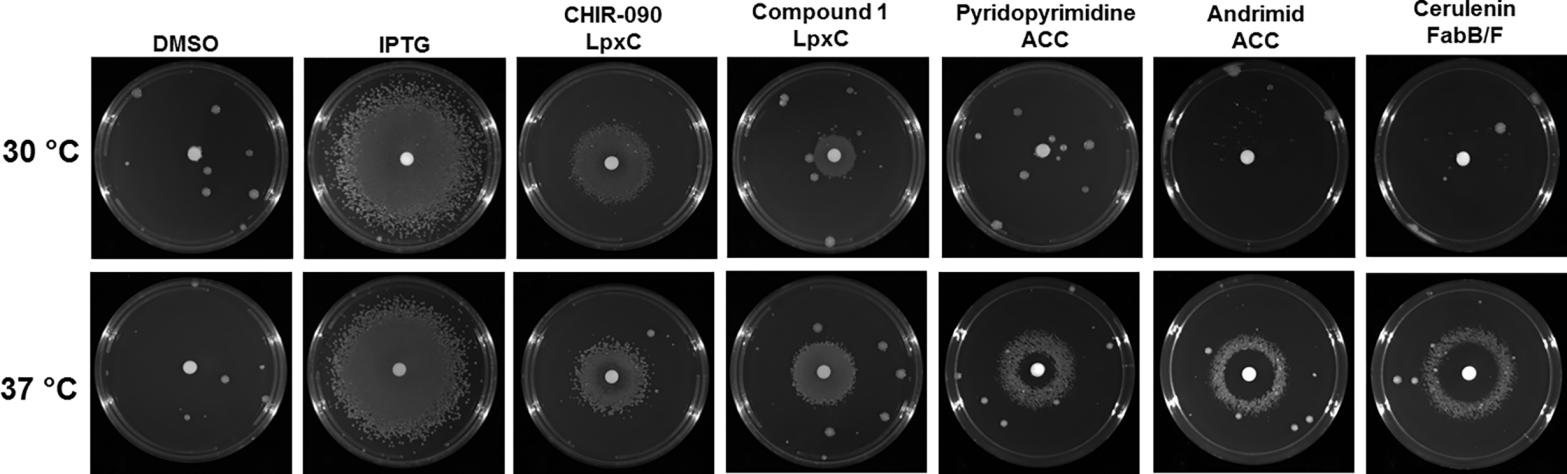
Inhibitors of fatty acid biosynthesis rescues growth of cells depleted for LpxK at 37°C but not 30°C. Growth of JWK0013(pNOV044) was not observed under noninducing conditions (DMSO, 10 μL per disk); growth of JWK0013(pNOV044) was restored in the presence of IPTG (10 μL @ 1 mM per disk) at 30 and 37°C; JWK0013(pNOV044) grew under noninducing conditions in the presence of CHIR-090 and Compound 1 (LpxC inhibitors, 10 μL @ 12.8 mg/mL) at 30 and 37°C; JWK0013(pNOV044) grew under noninducing conditions in the presence of fatty acid inhibitors at 37°C, but not 30°C, including pyridopyrimidine (10 μL @ 12.8 mg/mL per disk, ACC) and AFN-1252 (10 μL @ 3.2 mg/ml per disk, FabI) and cerulenin (10 μL @ 12.8 mg/mL per disk, FabB/F).

In *E*. *coli*, lethal defects in LPS transport can be suppressed by loss of *fabH* which causes a reduction in cell size and an increase in doubling time in order to slow the overall cell envelope growth which is believed to balance the biosynthesis of PL and LPS [87]. This rescue effect can be mimicked through a combined reduction in temperature (growth rate) and nutrient availability (size), suggesting that environmental factors can play a role in PL and LPS homeostasis [87]. Therefore, we asked whether temperature could affect the ability of the fatty acid and LPS inhibitors to rescue growth of *A*. *baumannii* ATCC 19606 under LpxK depletion conditions. At 37°C, a very clear zone of growth rescue occurred for both fatty acid and LPS inhibitors. However, only CHIR-090 and compound 1, a recently published hydroxamic acid LpxC inhibitor [20], were able to rescue growth at 30°C, demonstrating a differential growth rescue effect at 30°C compared to 37°C via these two pathways (Fig 3). Although the reason for this is not understood, it can serve as a convenient way to distinguish between inhibitors of the lipid A and fatty acid biosynthesis pathways.

### Proof of concept of utilizing the LpxK-controlled expression strain JWK0013(pNOV044) for the development of a whole cell positive growth screen to identity lipid A and fatty acid biosynthesis inhibitors

Our observations here that inhibitors of fatty acid biosynthesis or LpxC could rescue the growth defect of *A*. *baumannii* depleted for LpxK opened up the possibility of developing a cell-based, medium-throughput, positive-growth screen to identify novel small molecule inhibitors of fatty acid or lipid A biosynthesis. Inhibitors of lipid A biosynthesis (e.g. LpxA, LpxC, or LpxD) are not expected to be antibacterial in this strain background, and as such screening for those could be done at a single higher fixed concentration. However, inhibitors of fatty acid synthesis could be antibacterial at sufficiently high concentrations and therefore would only stimulate growth of the LpxK-depleted strain at sub-lethal concentrations high enough to provide sufficient inhibition of fatty acid synthesis to offset toxic accumulation of lipid A intermediates. From a practical standpoint it was therefore desirable to establish the screen in a dose-response format if both pathways were to be included, although this could be simplified to single point for inhibitors of lipid A biosynthesis.

**Fig 4:**
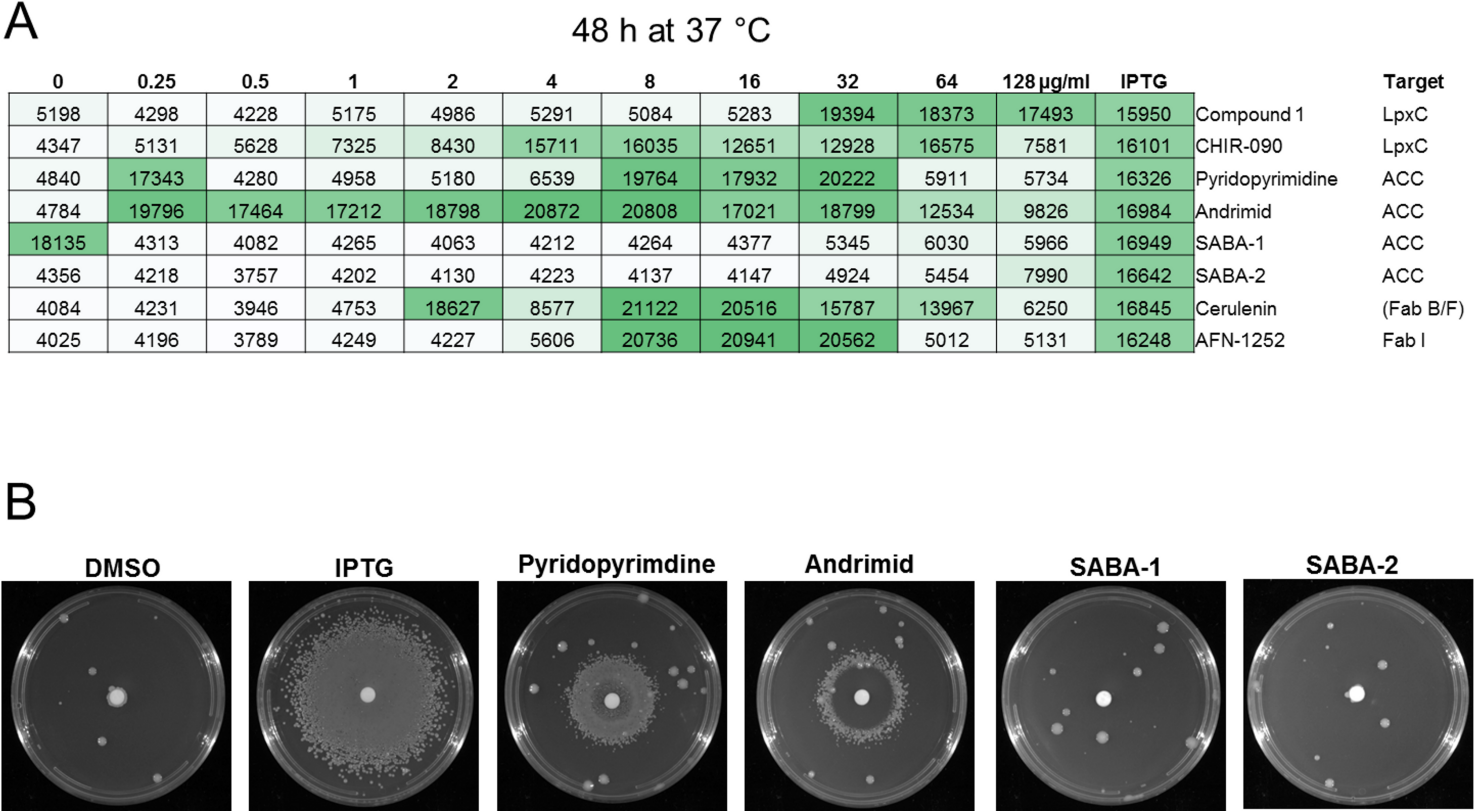
96-well growth restoration assay. A) Growth of JWK0013(pNOV044) was restored in the presence of LpxC inhibitors Compound 1 (32–128 μg/mL) and CHIR-090 (1–64 μg/mL) and fatty acid inhibitors pyridopyrimidine (8–32 μg/mL), andrimid (0.25–128 μg/mL), and cerulenin (2–64 μg/mL). Growth restoration was defined as an increase in fluorescence of at least 2-fold above background in consecutive wells. This was repeated at least three times in duplicate with similar results. In this representative example, SABA-1 displayed growth rescue at 0 μg/mL (DMSO control) due to revertants as evident by the lack of dose response, and growth was not observed in the replicate plates. B) Growth of JWK0013(pNOV044) was not observed under noninducing conditions (DMSO, 10 μL per disk); growth of JWK0013(pNOV044) was restored in the presence of IPTG (10 μL @ 1mM per disk); JWK0013(pNOV044) grew under noninducing conditions in the presence of pyridopyrimidine (10 μL @ 12.8 mg/ml per disk, ACC) and andrimid (10 μL @ 12.8 mg/ml per disk, ACC) but growth was not restored in the presence of SABA analogs that lack MIC values (10 μL @ 12.8 mg/mL per disk, SABA-1, 2).

Initial attempts to develop a 384-well high throughput screen in liquid were problematic due to poor growth of the chemically-rescued cells. Additionally, the high frequency of mutational loss of LpxK regulation and IPTG dependence (~1x10^-5^, also observed in the plate-based rescue experiments as noted by the presence of single colonies outside the zone of chemically rescued cells in Figs 2 and 3) led to a significant number of false-positive wells, as high as 10%, depending on the starting inoculum. Therefore, we developed a 96-well agar-based rescue assay to facilitate growth and limit interference by loss of *lpxK* regulation, as described in Methods. In this assay format, growth of JWK0013(pNOV044) was defined as an increase in fluorescence of at least 2-fold above background (DMSO only) in consecutive wells and was found to be restored in the presence of LpxC inhibitors CHIR-090 (2–64 μg/mL), Compound 1 (32–128 μg/mL) and fatty acid inhibitors cerulenin (2–64 μg/mL), pyridopyrimidine (8–32 μg/mL), AFN-1252 (8–32 μg/mL) and andrimid (0.25–128 μg/mL) (Fig 4A). We additionally tested sulfonamidobenzamide (SABA) analogs which have recently been shown to be inhibitors of ACC in Gram-negatives but lack whole cell activity in *E*. *coli* and *P*. *aeruginosa* [88]. Rescue of *A*. *baumannii* ATCC 19606 under LpxK depletion conditions was not observed in the presence of SABA-1 or SABA-2, which may be explained by the lack of MIC against *A*. *baumannii* and an inability to sufficiently inhibit the target at concentrations tested (Fig 4A and 4B, S2 Table). A growth rescue effect was not observed with levofloxacin, novobiocin, rifampicin, linezolid, A22, kanamycin, tobramycin, erythromycin, gentamicin, mecillinam, or meropenem S1 Fig.

## Discussion

The recent WHO report highlighting the Gram-negative pathogen *A*. *baumannii* (carbapenem-resistant) as a priority 1 (critical) pathogen underscores the urgency to identify new antibacterials for clinical development against this organism and other Gram-negative pathogens (http://www.who.int/mediacentre/news/releases/2017/bacteria-antibiotics-needed/en/). Historically, target based drug discovery efforts that begin with high-throughput biochemical screening against an essential target often fail to achieve potent whole cell activity and can be plagued by issues of cytotoxicity even after significant medicinal chemistry efforts are expended during lead optimization [3, 77, 78]. Ultimately, this process can be time-consuming and expensive, and only considers a fraction of the essential targets where biochemical assay development is feasible, as recently noted [8]. Alternatively, starting from a whole cell screening campaign where the target is unknown can also be resource intensive, to first identify the molecular targets of active compounds, and second to determine if the target itself is a desirable drug target [59]. For example, essential targets that are expected to require significant inhibition to produce a phenotype such as FabD, FabZ and FabA are thought to be undesirable for drug development within the FASII pathway [59]. As an alternative, pathway-directed whole cell screening approaches, as have been used to discover small molecule inhibitors of teichoic acid biosynthesis in *S*. *aureus*, may provide advantages including the ability to screen in the relevant organism, a simple assay format, the presence of biological activity, expectation of on-target activity, and the ability to target multiple enzymes simultaneously [89–92].

The observation that inhibition of LpxC, ACC, FabB/F and FabI could rescue the growth JWK0013(pNOV044) under noninducing conditions (without IPTG) provided an opportunity to evaluate the feasibility of developing a positive growth restoration screening assay to identity inhibitors of both fatty acid and lipid A biosynthesis in one assay. Furthermore, the number of nonspecific inhibitors (e.g. general membrane disruptive compounds) that typically comprise a significant percentage of the hits in growth inhibition based screens will be largely eliminated since this assay measures the restoration of growth rather than growth inhibition. However, it is also possible the screen could miss a genuine fatty acid inhibitor if the compound displays off target activity as was observed for triclosan. In this report, we provide proof of principle for the utilization of strain JWK0013(pNOV044) to identify inhibitors of lipid A and fatty acid biosynthesis by monitoring positive growth restoration. The assay relies on the antagonistic relationship inherently within LOS biosynthesis by leveraging the phenomenon of relieving toxic lipid A intermediate accumulation caused by depleting a late lipid A biosynthetic pathway step through inhibition of a second upstream biosynthetic enzymes to restore growth. We previously observed significant accumulation of DMSP (250-fold) and lipid X (40-fold) upon LpxK depletion in the regulated expression strain JWK0013(pNOV044) concomitant with cessation of growth [79]. Inclusion of the LpxC inhibitor CHIR-090 restored growth and reduced the levels of DSMP and lipid X to those typical of JWK0013(pNOV044) when LpxK expression was induced. Comparatively, LpxK depleted cells whose growth was rescued using the fatty acid inhibitor cerulenin still had somewhat elevated DSMP (25-fold) and lipid X (5-fold) which could explain the additional lag time in growth restoration [79]. To conclude, this assay can be utilized to complement high-throughput screening as part of downstream target identification efforts and is applicable to scaling through automation. With the continued focus on strategies to identify compounds that disrupt the permeability barrier, and the rapidly expanding ability to screen new chemical matter, assays that effectively and cheaply enable mode of action or target identification are expected to lead to quicker decision making in identifying lead molecules for development.

## Supporting information

S1 Fig 96-well growth restoration assay. **A)** Growth was not restored in the presence of levofloxacin, novobiocin, rifampicin, linezolid, A22, meropenem, or mecillinam. B) Growth of JWK0013(pNOV044) was not restored in the presence of kanamycin, gentamicin, tobramycin, rifampicin, meropenem, erythromycin, azithromycin, levofloxacin, linezolid, novobiocin and mecillinam.
(PDF)

S1 Table Antibiotics used in this study. (PDF)

S2 Table Antibiotic susceptibilities of *A*. *baumannii* strains (μg/ml). (PDF)
