# Supplementary material for: A pathway-directed positive growth restoration assay to facilitate the discovery of lipid A and fatty acid biosynthesis inhibitors in Acinetobacter baumannii
Source: PLoS One. 2018 Mar 5;13(3):e0193851. doi: 10.1371/journal.pone.0193851 (PMC5837183; doi:10.1371/journal.pone.0193851)

A

0	0.25	0.5	1	2	4	8	16	32	64	128 µg/ml	IPTG		Target
6819	6411	5891	6161	5862	6020	5915	6039	6178	6551	6785	16425	Levofloxacin	Gyrase
6871	17391	6668	7013	6432	6352	6029	5957	6236	6792	7225	16012	Novobiocin	Gyrase
6804	6484	6161	6041	5497	5414	5190	4451	4375	4940	4792	16289	Rifampicin	Ribosome
6925	19426	6662	7257	17454	6483	6341	6238	6431	7290	7727	16115	Linezolid	Ribosome
7019	6771	6889	6908	6860	6688	6693	6520	7222	7621	8104	15937	A22	MreB
7030	6443	6866	7060	7058	6690	7252	7243	7924	9299	11170	15833	Meropenem	PBP
7082	6537	6413	7137	7407	18414	7470	7484	7754	8216	8526	15333	Mecillinam	PBP
7234	6697	8227	15476	20017	17333	15516	15949	16768	15549	9250	14156	CHIR-090	LpxC

B

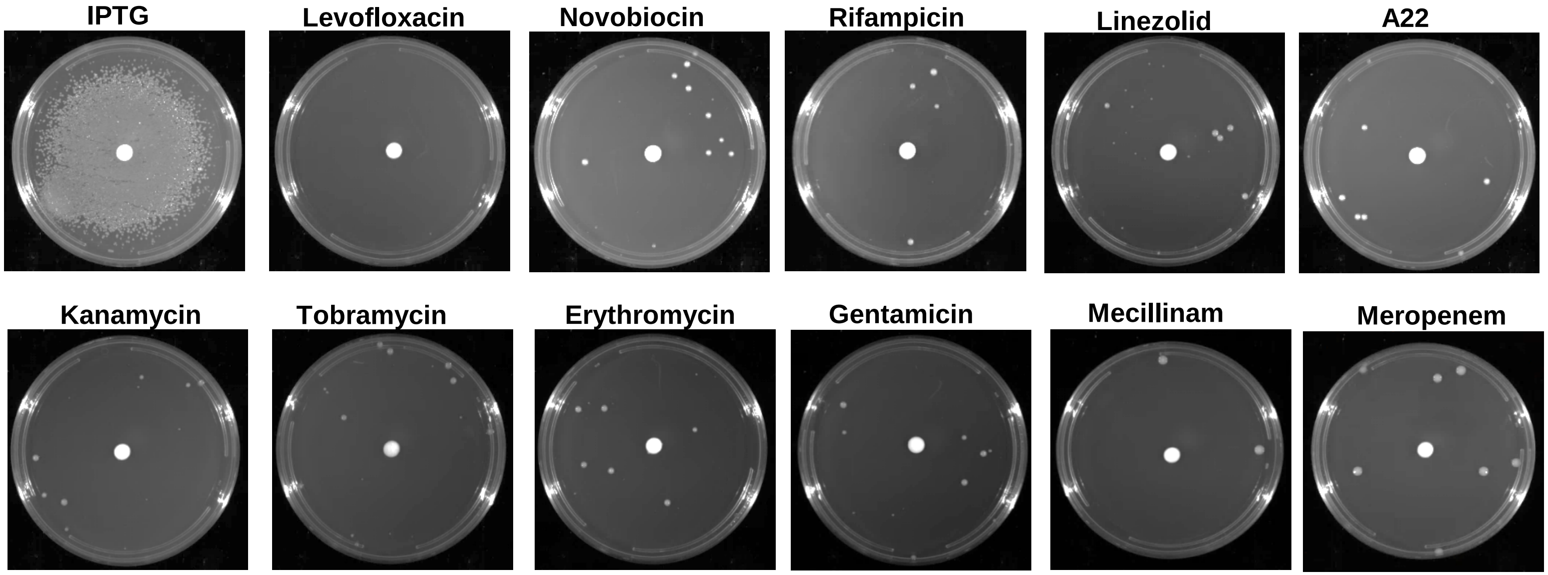

Supplement: S1 Fig — A) Growth was not restored in the presence of levofloxacin, novobiocin, rifampicin, linezolid, A22, meropenem, or mecillinam. B) Growth of JWK0013(pNOV044) was not restored in the presence of kanamycin, gentamicin, tobramycin, rifampicin, meropenem, erythromycin, azithromycin, levofloxacin, linezolid, novobiocin and mecillinam. (PDF) [file pone.0193851.s001.pdf]
