# Supplementary material for: A pathway-directed positive growth restoration assay to facilitate the discovery of lipid A and fatty acid biosynthesis inhibitors in Acinetobacter baumannii
Source: PLoS One. 2018 Mar 5;13(3):e0193851. doi: 10.1371/journal.pone.0193851 (PMC5837183; doi:10.1371/journal.pone.0193851)
Supplement: S1 Table — (PDF) [file pone.0193851.s002.pdf]

Antibiotics	Source	Catalog number
Triclosan	USP	68220
Cerulenin	Enzo	BML-G237-0005
SABA-1	ChemBridge Corp	7106106
SABA-2	ChemBridge Corp	7478794
Compound 1 (20)	Novartis Compound Library	
Pyridopyrimidine	Novartis Compound Library	
CHIR-090	Axon MedChem	Axon 2000
AFN-1252	MedChem Express	HY-16911
Andrimid	Novartis Compound Library	
Kanamycin	Sigma	K-1637
Gentamicin	Sigma	G-3632
Tobramycin	USP	1667508
Erythromycin	Sigma	E8755
Azithromycin	USP	1046056
Levofloxacin	Sigma	28266-IC-F
Novobiocin	Sigma	N-6160
Rifampicin	Sigma	R-3501
Linezolid	Novartis Compound Library	
A22	Sigma	SML0471
Meropenem	USP	1392454
Mecillinam	Alfa Aesar	566774-ML
