# Supplementary material for: A pathway-directed positive growth restoration assay to facilitate the discovery of lipid A and fatty acid biosynthesis inhibitors in Acinetobacter baumannii
Source: PLoS One. 2018 Mar 5;13(3):e0193851. doi: 10.1371/journal.pone.0193851 (PMC5837183; doi:10.1371/journal.pone.0193851)
Supplement: S2 Table — (PDF) [file pone.0193851.s003.pdf]

Antibiotic / (Target)	Strain		
	ATCC 19606	*lpxC::Km*^{R}	*lptD::Km*^{R}
AFN-1252 (FabI)	32 - > 32	0.125 - 0.5	0.0156
Andrimid (ACC)	64	1	< 0.25
Cerulenin (FabF/B)	32-64	16	2
Pyridopyrimidine (ACC)	128	16 - 32	8-16
CHIR-090 (LpxC)	64	128	128
Compound 1 (LpxC)	>128	>128	>128
SABA-1 (ACC)	>128	16	4-8
SABA-2 (ACC)	>128	64	16 - 32
Triclosan (FabI)	0.125 - 0.25	0.25	< 0.0019
